# A Multi-Platform Flow Device for Microbial (Co-) Cultivation and Microscopic Analysis

**DOI:** 10.1371/journal.pone.0036982

**Published:** 2012-05-14

**Authors:** Matthijn C. Hesselman, Dorett I. Odoni, Brendan M. Ryback, Suzette de Groot, Ruben G. A. van Heck, Jaap Keijsers, Pim Kolkman, David Nieuwenhuijse, Youri M. van Nuland, Erik Sebus, Rob Spee, Hugo de Vries, Marten T. Wapenaar, Colin J. Ingham, Karin Schroën, Vítor A. P. Martins dos Santos, Sebastiaan K. Spaans, Floor Hugenholtz, Mark W. J. van Passel

**Affiliations:** 1 Wageningen UR iGEM 2011 Team, Wageningen University, Wageningen, The Netherlands; 2 MicroDish BV, Utrecht, The Netherlands; 3 Food Process Engineering, Wageningen University, Wageningen, The Netherlands; 4 Systems and Synthetic Biology, Wageningen University, Wageningen, The Netherlands; 5 Laboratory of Microbiology, Wageningen University, Wageningen, The Netherlands; 6 Netherlands Consortium for Systems Biology, University of Amsterdam, Amsterdam, The Netherlands; University of Hyderabad, India

## Abstract

Novel microbial cultivation platforms are of increasing interest to researchers in academia and industry. The development of materials with specialized chemical and geometric properties has opened up new possibilities in the study of previously unculturable microorganisms and has facilitated the design of elegant, high-throughput experimental set-ups. Within the context of the international Genetically Engineered Machine (iGEM) competition, we set out to design, manufacture, and implement a flow device that can accommodate multiple growth platforms, that is, a silicon nitride based microsieve and a porous aluminium oxide based microdish. It provides control over (co-)culturing conditions similar to a chemostat, while allowing organisms to be observed microscopically. The device was designed to be affordable, reusable, and above all, versatile. To test its functionality and general utility, we performed multiple experiments with *Escherichia coli* cells harboring synthetic gene circuits and were able to quantitatively study emerging expression dynamics in real-time via fluorescence microscopy. Furthermore, we demonstrated that the device provides a unique environment for the cultivation of nematodes, suggesting that the device could also prove useful in microscopy studies of multicellular microorganisms.

## Introduction

In recent years there have been numerous attempts to develop novel platforms for growing previously uncultured microbes, and to expand the scope and precision of the study of bacterial model organisms. While the vast majority of bacterial and archaeal species remain uncultivable, recent studies have shown that an increasing number of environmental isolates can be grown in artificial environments that mimic the physical and chemical parameters of the organisms' natural habitat [1], [2], [3]. Conversely, quantitative, systems-oriented investigations of highly engineered model organisms containing synthetic regulatory networks benefit from experimental set-ups which allow precise control to be exerted over these same parameters, while physiological responses are monitored in real time without disrupting the cells [4], [5], [6]. These platforms range from simple do-it-yourself approaches to more high-tech methods [7], [8], [9]. While some of these approaches, which include microfluidic chips [10], porous metallic membranes [2], and gel microdroplets [11], among others, vary greatly in terms of scale, material, and level of sophistication, they share a number of common characteristics. In all cases the microorganisms are physically trapped, and thereby constrained in terms of mobility and population size. Nutrients are either provided through direct channels, or via diffusion across the trapping barrier. Optical transparency of the materials is also common, as it facilitates microscopic observations.

**Figure 1:**
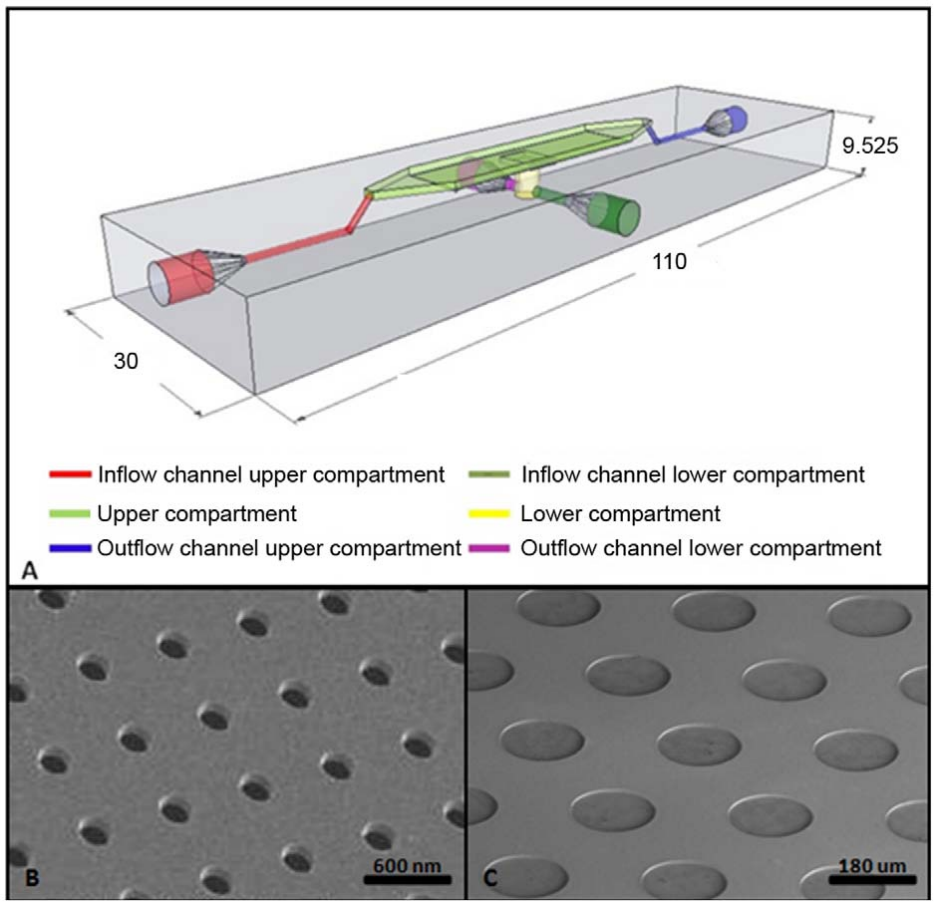
Schematic representation of the flow device. A) Schematic representation of the flow device, with the dimensions in mm. Depicted in red and blue are the in- and outflow channels of the top compartment (light green). The respective in- and outflow channels of the lower compartment (yellow) are given in purple and dark green. B) Electron microscopy image of a microsieve. C) Electron microscopy image of a microdish. See File S1 for more views of the device.

**Table 1:** Overview of the BioBrick parts used.

Experimental set-up	BioBrick part	Reference
Variations in signal strength over time	BBa_K546546	http://partsregistry.org/Part:BBa_K546546
**Co-cultivation:**		
Inducible cells	BBa_K546002	http://partsregistry.org/Part:BBa_K546002
Inducer cells*	BBa_K546000	http://partsregistry.org/Part:BBa_K546000
	BBa_I721007	http://partsregistry.org/Part:BBa_I721007

* co-cultivated with RFP containing BioBrick part as control for leakage.

Elegant microfluidic cultivation devices may not only reduce costs, but can also increase the accuracy and precision of measurements, enabling new experimental approaches and testing novel hypotheses [12]. Still, microfluidic devices frequently require sophisticated and expensive peripheral equipment, potentially complicating the simplest of analyses. Furthermore, microfluidic devices are vulnerable to fouling and contamination when used to deliver culture medium, which reduces the robustness of such methods and their usable lifetime.

Within the context of iGEM, the international Genetically Engineered Machine competition [13], a team of undergraduate students from Wageningen University re-designed a genetic circuit for synchronized oscillations, inspired by previous studies in *Escherichia coli* [4], [6]. To study the dynamics of synchronized oscillatory gene expression, a simple – and by all means affordable–bacterial cultivation platform was required, in which quantitative fluorescence measurements on small bacterial populations could be performed. Previous work on synchronized bacterial oscillators has demonstrated the importance of providing growth conditions in which high-density, spatially constrained bacterial populations can be nutritionally sustained over prolonged periods of time [4]. Attempts to observe synchronized oscillatory behavior using conventional experimental set-ups, such as microtiter plates, proved irreproducible. In order to provide the necessary conditions, we envisioned a simple growth chamber in which small microbial populations could be immobilized and which could be operated in conjunction with a fluorescence microscope taking measurements in real time, with medium flow supplied by a standard syringe pump.

To this end, we designed, manufactured and tested a simple and reusable plastic flow chamber with a custom socket that can accommodate two different cultivation platforms. One of the cultivation platforms in this flow chamber is a silicon nitride microsieve that is available with a range of different pore sizes, which has its origins in microfiltration applications (http://www.aquamarijn.nl/). The second platform is a microbial culture chip (microdish) made of porous aluminium oxide (PAO) (http://www.microdish.nl/) [2]. The flow device enabled us to monitor the activity of fluorescent reporter genes from microbes under the microscope, while providing control over the supply of growth media without mechanically disrupting the cells.

## Materials and Methods

### Design and construction of the multi-platform flow device

The device is made from polymethylmethacrylate (perspex), a transparent, durable, autoclavable, and inexpensive material (Figure 1). The in- and outlets are threaded, which allows plugs and tubing to be screwed in securely. The material is resistant to ethanol and chlorine-based cleaning solutions, and can be cleaned with these to sterilize the device between experiments (http://www.gehr.de/dyndata/PMMA_engl.pdf). The device is not as susceptible to biofouling as microfluidic chips due to the internal dimensions that allow cleaning solutions to remove any biological residue. We did not observe any residual contamination after repeated experiments (data not shown). The distance between the socket and the top of the chamber is ∼1 mm. This proximity allows for a magnification with a 20x objective for a total magnification of 200x using an Olympus Microscope BX41, which allows the detection of fluorescence emitted by small agglomerations of cells. Detection of single bacterial cells may be also possible using objectives with higher magnification as long as the minimum focal depth is no less than 1 mm, such as 40x and 60x water-immersion objectives of the Olympus LUMPLFLN-W series. The dimensions of the chamber were chosen such that both growing platforms could be accommodated. The resulting total volume of the main chamber is ∼0.5 ml. The device can be sealed with a thin glass slide using Bison mastic silicon kit, which is removable and enables the reuse of the device after an experiment. The flow device was used with a ProSense syringe pump NE1000X2 for the flow of media or buffers which is connected to the device via transparent silicon tubing. The flow device was designed using Google SketchUp (http://sketchup.google.com), and manufactured with a standard workbench computer controlled drill in the fine mechanical workshop of Wageningen University. The sketch files of the device are available for users to customize and manufacture their own flow device (File S1). The cost of a single, reusable device, excluding a microsieve or microdish, can be approximated to be <150 Euros, the bulk of which can be attributed to manual labor costs.

**Figure 2:**
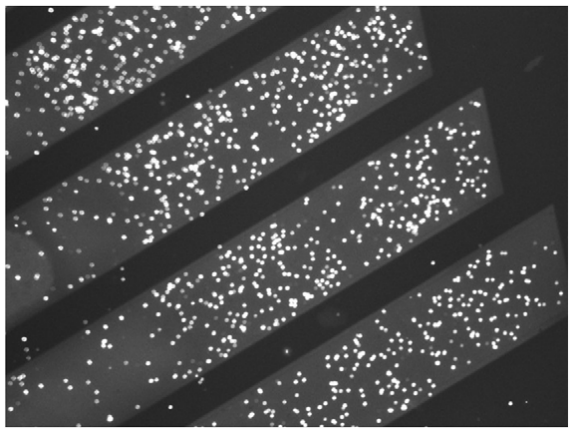
Cell retention on the microsieve. Retained clusters of fluorescent *E. coli* cells on the sieve (100x magnification). The diagonal light grey bands are the permeable areas of the microsieve that contain the pores.

### Microsieve cultivation

Microsieves are inorganic membranes made of a thin layer of silicon-rich silicon nitride [14]. The microsieve comes in a wide range of variants with respect to different well-defined pore sizes (0.2 µm–0.45 µm), thicknesses (0.1–1 µm) and different levels of porosity. The silicon nitride has high thermal stability, chemical inertness and mechanical strength. Together this allows high flux performance with low trans-membrane pressure and size-selectivity [15], [16], [17], [18].

**Figure 3:**
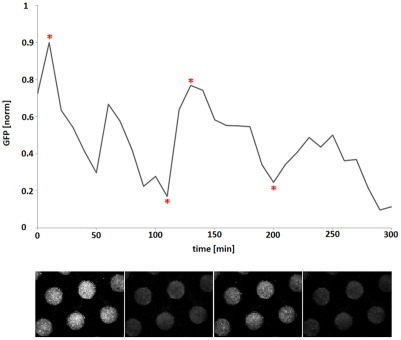
Oscillating GFP expression observed in microdish wells. Fluorescent *E. coli* cells in the wells of the microdish showing variations in signal strength over time. The graph depicts variations plotted with the image analysis and processing tool ImageJ. The x-axis represents time, and the y-axis represents fluorescence (in arbitrary units and with a variance of maximally 0.01 for the normalised data of 5 wells). Below the graph are microscopic images of fluorescent bacteria in the cultivation chip wells at different intervals using identical illumination conditions and CCD camera exposure times. The time points at which the images were taken are indicated with an asterisk.

To retain fluorescent *Escherichia coli* cells on the microsieve (pore size 0.45 µm), the cells were inoculated through the top inlet channel while applying negative pressure below the sieve to partly remove the liquid and retain the bacteria on the sieve. This was done manually with the use of a syringe attached via plastic tubing to one of the lower channels.

### Microdish cultivation

The material (PAO) used for the microdish is a broadly applicable and modifiable matrix enabling an increased cultivation efficiency, for example of pathogens [19], [20], and is available with and without circular microscopic subdivisions into micron scale wells, which can serve as growth chambers. The microdish comes in a wide range of variants with respect to the presence and absence of subcompartments, subcompartment dimensions, chemical modifications of the PAO, but also with respect to the thickness and pore size and porosity of the cultivation chip itself. Together this allows for a range of dynamics with respect to the diffusion rate of small molecules and the ability to passage or exclude macromolecules.

**Figure 4:**
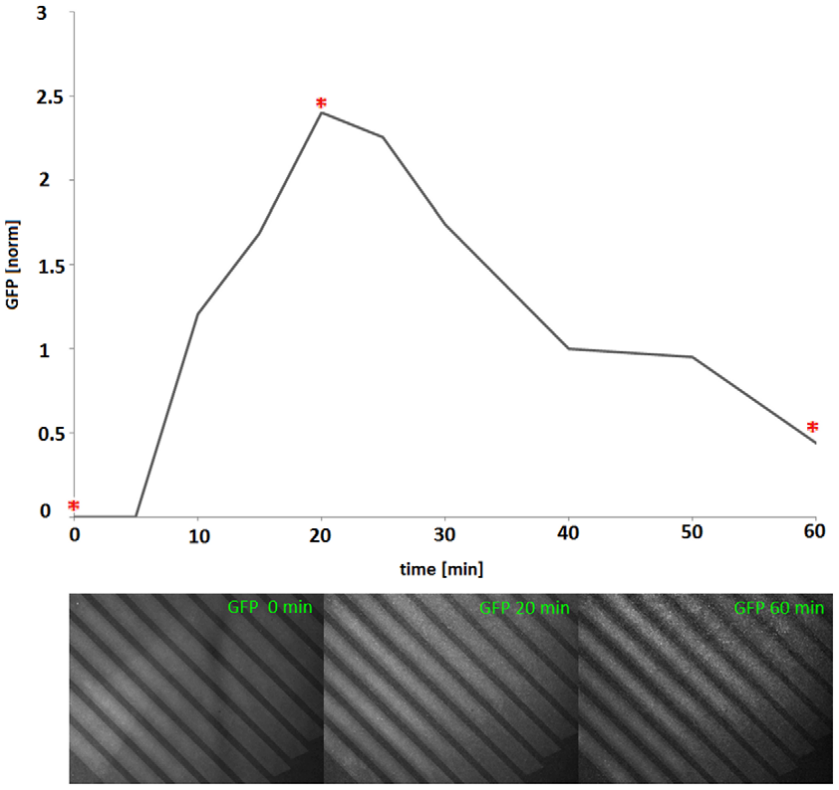
Co-cultivation of cells separated by a microsieve. Increase of GFP expression of inducible cells on the sieve after inoculation of inducer cells below. Graph plotted with the image analysis and processing tool ImageJ. The x-axis corresponds to time and the y-axis shows the detected GFP signal (in arbitrary units). Below: a number of representative images of the microsieve. The time points at which the images were taken are indicated with an asterisk.

**Figure 5:**
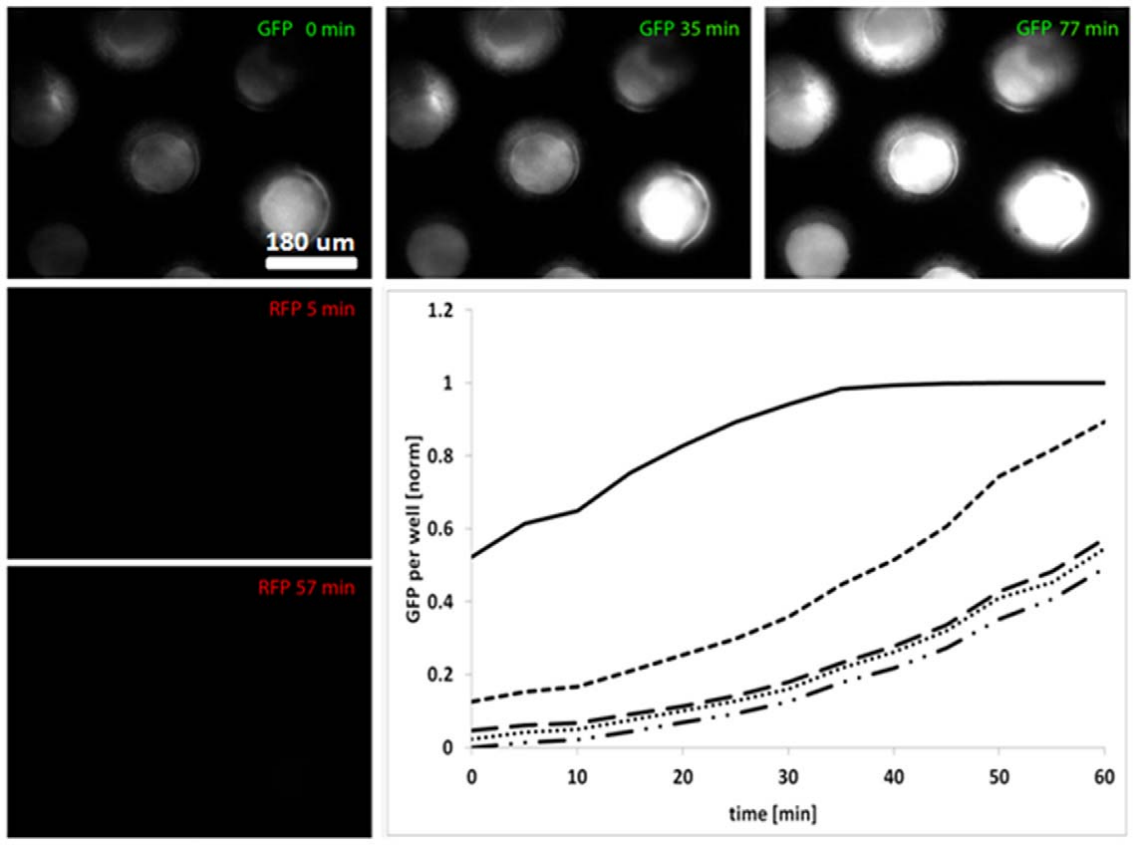
Co-cultivation of cells in the flow device. Expression of GFP increases over time in cells growing in microdish wells after adding inducer (Acyl homoserine lactone (AHL) producing) and RFP expressing cells to the bottom compartment of the flow device. The intensity of the light emitted from five wells was quantified using imageJ and normalized against the background. No RFP was detected in the top chamber, indicating that the inducer-cells added to the bottom chamber did not come in contact with the top, and GFP expression was induced by diffusion of the inducer (AHL) through the microdish.

A compartmentalized microdish cultivation chip, containing 40 µm deep wells with a diameter of 180 µm, was tested in the flow device. An overnight-grown *E. coli* culture containing a synthetic gene construct capable of producing synchronized oscillatory gene expression was resuspended in phosphate buffered saline (PBS). The resuspended culture was used to inoculate the device through the top inlet channel. LB medium was supplied via the lower inlet channel, thus restricting the growth of bacteria to the wells where nutrients could be obtained through the porous material at the base. Several methods that allow wells to be individually inoculated have been described [21], [22], [23]. However, these specialized inoculation techniques were not necessary for the experiments performed during the validation of our device. Using an Olympus BX41 microscope equipped with a GFP filter, variations in fluorescent light emission were measured. The light source was a HBO 103W/2 Osram mercury lamp. Images were captured by a CCD camera with an exposure time of 200 ms. Cells were illuminated for 2 seconds for every measurement. The shutter was robotically controlled to ensure consistent illumination at each time point. Data analysis and processing were done with the software ImageJ 1.45 (http://rsbweb.nih.gov/ij/index.html). Additionally, experiments to test the applicability of co-cultivation in the microsieve and microdish were performed. For this, cultures of inducer and inducible cells were grown separately overnight. The inducible cells were inoculated in the top compartment of the flow chamber containing a microsieve, followed shortly thereafter by the inoculation of inducer cells in the lower compartment. For the same experiment in the microdish, the inducible cells were resuspended in PBS before inoculating via the top inlet channel. LB media was supplied via the lower inlet channel to enable the cells to grow in the wells. After letting the inducible cells grow in the dish for one night, the inducer cells were injected into the lower compartment. In both experimental set-ups, GFP measurements of the top cells were taken in short intervals (5–10 minutes) directly after inoculation of the inducer cells.

### Organisms and constructs

The bacteria used for the experiments were *E. coli* TOP10 cells (Invitrogen) with a pSB1A2 plasmid backbone encoding ampicillin resistance (http://partsregistry.org/Part:pSB1A2) and containing different BioBrick parts as they can be found in the Registry of Standard Biological Parts (http://partsregistry.org). BioBrick standard biological parts are DNA sequences that adhere to defined standards to facilitate the modular assembly of complex genetic constructs from simple subcomponents. The principle feature of BioBrick parts is the presence of unique restriction sites flanking the functional DNA elements [24]. The parts used are depicted in table 1.

The liquid cultures were made from single colonies from cell cultures grown on LB agar plates with ampicillin (50 µg/mL). These were grown over night at 37°C in 10 ml of LB medium containing ampicillin. The cultures were either directly inoculated in the device or centrifuged and resuspended in PBS, depending on the experimental set-up.

To assess whether the flow device could be used for other organisms, an engineered *Caenorhabditis elegans* strain PD4792 expressing green fluorescent protein (GFP) exclusively in the oesophagus (www.wormbase.org) was injected into the top chamber. The fluorescence allows for easier detection of the nematodes, but the animals are also visible with white light from above. In an additional experiment, the wells were first seeded with green fluorescent protein producing *E. coli*, serving as a food source for the animals. The nematodes were placed in the upper compartment the next day and the liquid over the wells was removed. Nematodes that remained trapped in the wells were left in the dish for another night.

## Results and Discussion

### Retention of bacteria on a microsieve

The device containing a microsieve was inoculated with fluorescent *E. coli* cells. The bacteria were retained predominantly on the diagonal permeable areas, where small clusters of cells could be discerned. Even after applying a gentle flow over the retained cells, potentially allowing exposure of the cells to other compounds in the context of biosensing, the cells remained trapped on the sieve (Figure 2).

**Figure 6:**
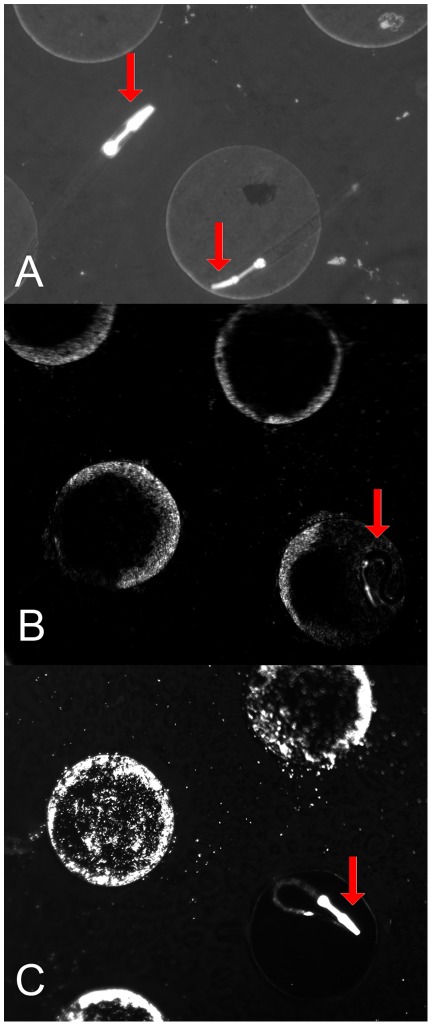
Fluorescent nematodes observed in the flow device. A) Nematodes floating over the wells while the chamber is filled with liquid. The fluorescent oesophagus in the front side of the nematode is clearly visible. B) Nematode trapped in a well filled with fluorescent *E. coli* cells after removing the liquid from the chamber. C) Next day: A nematode after consuming all fluorescent bacteria from the well, resulting in observable fluorescence in the nematode intestine.

### Synchronized oscillatory gene expression in a microdish

Certain intercellular signalling networks consisting of regulatory feedback loops can result in synchronized oscillatory gene expression. However, the emergence of such phenomena is generally not very robust, and often contingent on specific environmental parameters. Temporal variations in GFP expression produced by *E. coli* microcolonies harboring such a signalling construct were detected using fluorescence microscopy. The averaged and normalized fluorescent emission intensities plotted against time clearly depict synchronized oscillatory behavior with a period of approximately 1 h (Figure 3). Since the aim of this experiment was to detect relative changes in fluorescence intensities over time in order to confirm the oscillatory properties of the construct rather than measurement of absolute GFP expression values, it was not necessary to normalize the measured intensities against the cell density.

### Co-cultivation experiments

In nature, most microorganisms grow in co-culture with other microbes, and interactions mediated by diffusible signalling compounds are critical in this process and often essential for growth [1], [3]. Relatively simple co-cultivation systems based around porous membranes have led to major improvements in the cultivation of otherwise intractable species. In the laboratory, co-cultivation experiments are comparatively rare and usually confined to defined pairs of microorganisms.

The separation of the flow chamber into two compartments by either of the two cultivation platforms allows the co-cultivation of different organisms separated by the permeable platform, thereby enabling an exchange of small molecules, for example in co-dependent cross-feeding experiments [25]. Alternatively, since PAO also allows for eukaryotic cell culturing [26], this flow device could allow for experiments on growth promotion, infectivity or toxicity.

To test the applicability of the set-up for co-cultivation, we assessed whether a signalling molecule produced by bacteria in the lower compartment would induce gene expression of bacteria in the top compartment. The signalling molecule produced by the bacteria in the lower compartment (inducer cells) should easily diffuse through the permeable platform and be bound by a receptor present in the bacteria in the top compartment (inducible cells). The resulting complex in turn activates gene expression of a detectable reporter, in this case GFP. The experiment was carried out in flow devices equipped with either a microsieve or a microdish.

Initial measurements of the inducible cells in the top compartment showed hardly detectable amounts of basally expressed GFP. After addition of the inducer culture in the lower compartment, a rapid increase of GFP expression was observed, both in the microsieve (Figure 4) and the microdish (Figure 5). The observed increase was too rapid to be the result of cell growth alone. This can be concluded from the observation that cells constitutively expressing GFP do not display increases in fluorescence of a comparable rate and magnitude (data not shown). In order to validate the barrier function of the two platforms, the suspension of inducer cells was spiked with *E. coli* cells expressing RFP. Additionally to the GFP measurements, the top compartment was monitored for presence of RFP. Leakage was not observed, indicating that the edges of the respective growing platforms were sealed properly.

The system presented here would allow the gap between nature and the laboratory to be bridged, with thousands of different microorganisms (in discrete microwells) to be co-cultivated with a common partner organism (beneath the microdish), a major increase in combinations that may be further improvable with higher well densities to allow genuinely high throughput screenings for microbial interactions.

### Using the flow device with nematodes

The device could be useful for the cultivation of organisms other than bacteria, for example for *Caenorhabditis elegans*, a nematode approximately 1 mm in length and 80 µm in width. The nematodes inoculated in the upper compartment of a flow device containing a microdish were easily discernible (Figure 6), and capable of moving over the wells in the dish when the top chamber of the device was filled with liquid. Cultivation experiments on PAO indicated that the full developmental cycle, from eggs to adult, could occur on this material (data not shown). The pictures taken one day after inoculation revealed that the wells containing worms were almost completely devoid of the bacteria, while wells without the animals were still filled with the fluorescent *E. coli* cells (Figure 6). This highlights a potential use of the flow device with multicellular organisms. Approaches to the analysis of nematodes using microfluidics have used relatively complicated platforms requiring multistep fabrication techniques. Furthermore, these approaches have been limited in throughput, resulting from the need to supply individual chambers with input and output channels [10], [27], [28]. Given the importance of nematodes in drug screening, e.g. drugs targets against the central nervous system, robust high-throughput methods are desirable and the method presented here provides a route to one, particularly given the potential to add drugs either from beneath the culture chip or by non-contact printing into individual wells or by co-culture with bacterial strains expressing siRNAs.

### Conclusions

The recent development of novel microbial cultivation platforms has provided researchers with a wide variety of options for isolating and studying microorganisms. It is important to be aware of the advantages and drawbacks of the available platforms. Factors such as population size, geometry, control over medium composition, parallelization and multiplexing capacity need to be weighed against the cost of, and the expertise and peripheral equipment required to operate a given platform. The most basic implementation of what can be considered a novel cultivation platform is a simple matrix, which combines cell adhesion with nutrient diffusion. Such set-ups are inexpensive, and easy to implement as they do not require sophisticated peripheral equipment. On the other end of the spectrum, there are nanoscale microbioreactor chambers, which allow individual cells to be subjected to precisely controlled chemical environments [7]. The microbial flow device described in this paper is somewhere in between. It is well suited for fluorescence microscopy studies of small, high-density cellular populations over extended time periods, as it provides control over the composition of liquid media without disrupting the cells. We have demonstrated its utility in the study of intercellular signalling, dynamic gene regulatory networks, and nematode cultivation. Other potential applications of this device include microbial biosensing, toxicity measurements and chemotaxis studies. Furthermore, since the platforms used in this device allow for the cultivation of eukaryotic cells, it could be of use in the real-time monitoring of co-cultivation and infection experiments.

We believe that this device has the potential to make advanced cultivation materials accessible to a wider range of users. While we do not anticipate that this platform will replace existing technologies, we hope that our design, and modifications thereof, will prove useful where versatility, cost, and simplicity of use need to be emphasized over experimental precision, such as in the DIY biology community and in iGEM projects.

## Supporting Information

File S1 **Schematic representations of the flow device, with the dimensions in** µm**.** Depicted in blue and red are the in- and outflow channels of the top compartment (light green). The microdish socket is shown in grey, and the microsieve socket is shown in dark purple. The respective in- and outflow channels of the lower compartment (yellow) are given in light purple and dark green. A) Side view of the flow device without external framework. B) Top view of the flow device without external framework. C) Zoomed in top view of the flow device including microsieve and microdish socket dimensions. D) Top view of top compartment of the flow device including top compartment dimensions. E) Bottom side view flow device without external framework.
(DOCX)
