# Supplementary material for: A Multi-Platform Flow Device for Microbial (Co-) Cultivation and Microscopic Analysis
Source: PLoS One. 2012 May 14;7(5):e36982. doi: 10.1371/journal.pone.0036982 (PMC3351485; doi:10.1371/journal.pone.0036982)
Supplement: File S1 — Schematic representations of the flow device, with the dimensions in µm. Depicted in blue and red are the in- and outflow channels of the top compartment (light green). The microdish socket is shown in grey, and the microsieve socket is shown in dark purple. The respective in- and outflow channels of the lower compartment (yellow) are given in light purple and dark green. A) Side view of the flow device without external framework. B) Top view of the flow device without external framework. C) Zoomed in top view of the flow device including microsieve and microdish socket dimensions. D) Top view of top compartment of the flow device including top compartment dimensions. E) Bottom side view flow device without external framework. (DOCX) [file pone.0036982.s001.docx]

Supplementary file S1 **Schematic representations of the flow device**

Schematic representations of the flow device, with the dimensions in µm. Depicted in blue and red are the in- and outflow channels of the top compartment (light green). The microdish socket is shown in grey, and the microsieve socket is shown in dark purple. The respective in- and outflow channels of the lower compartment (yellow) are given in light purple and dark green. A) Side view of the flow device without external framework. B) Top view of the flow device without external framework. C) Zoomed in top view of the flow device including microsieve and microdish socket dimensions. D) Top view of top compartment of the flow device including top compartment dimensions. E) Bottom side view flow device without external framework.


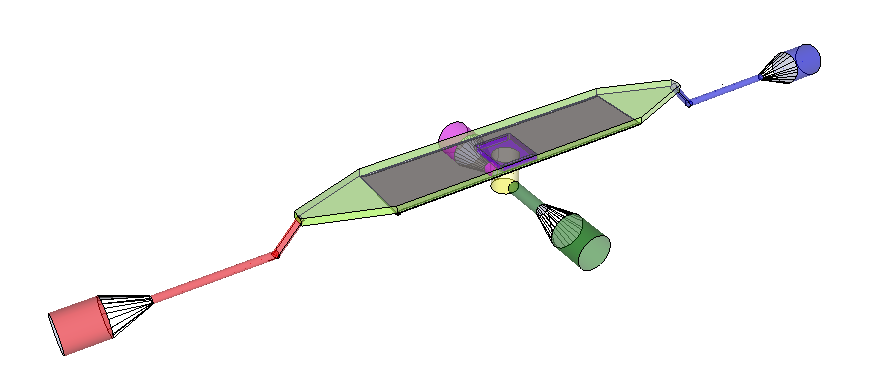


**A**


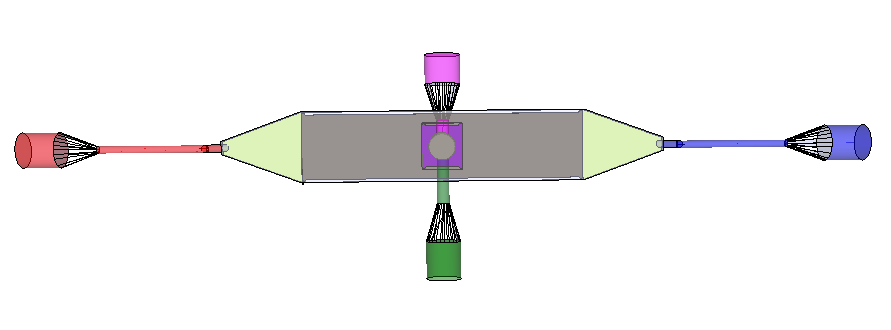


**B**


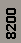

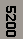

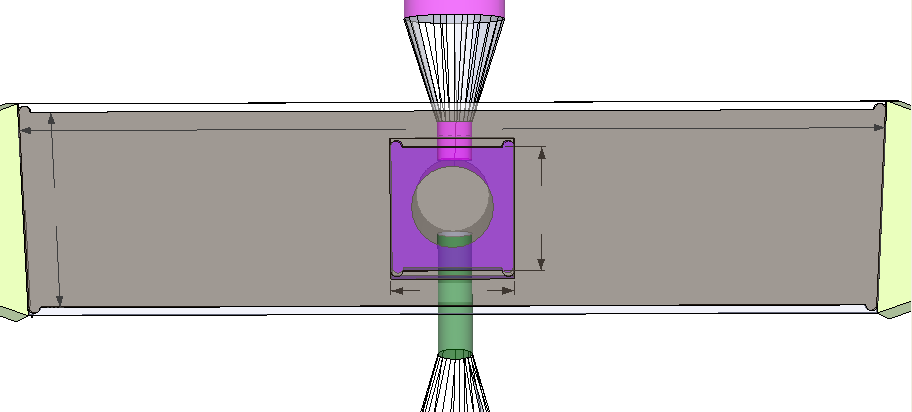


**C**

**35800**

**5200**


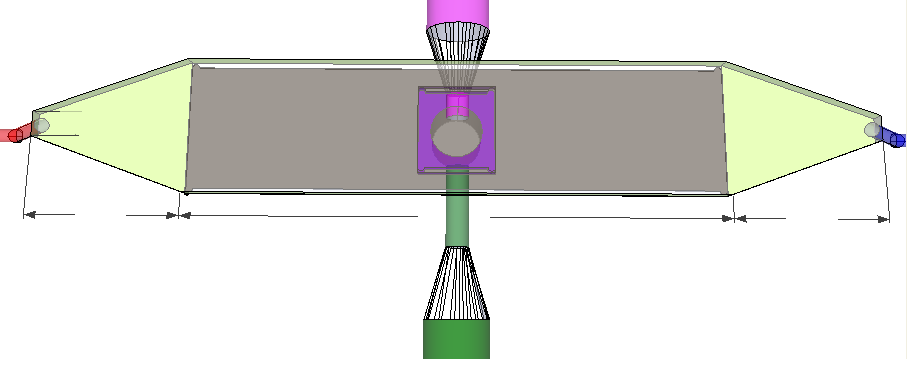

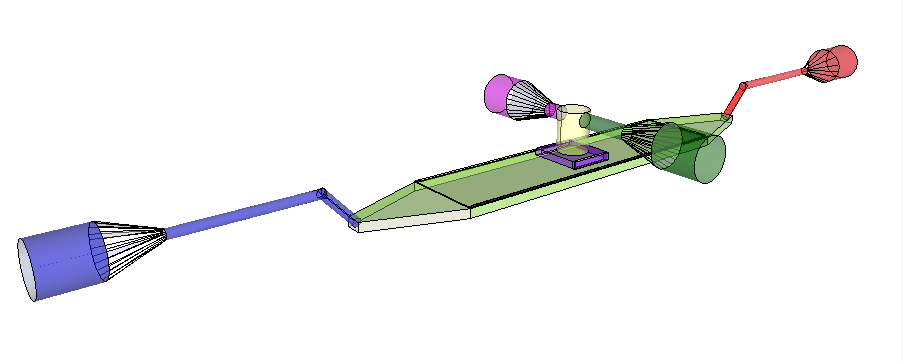


**E**

**D**

**10000**

**35800**

**10000**

**1650**
